# A Local Community-Based Social Network for Mental Health and Well-being (Quokka): Exploratory Feasibility Study

**DOI:** 10.2196/24972

**Published:** 2021-10-27

**Authors:** Cynthia Shih, Ruhi Pudipeddi, Arany Uthayakumar, Peter Washington

**Affiliations:** 1 Quokka Palo Alto, CA United States; 2 Department of Computer Science University of California, Berkeley Berkeley, CA United States; 3 Department of Cognitive Science University of California, Berkeley Berkeley, CA United States; 4 Department of Bioengineering Stanford University Stanford, CA United States

**Keywords:** local social network, community health, wellbeing, digital health, consumer health

## Abstract

**Background:**

Developing healthy habits and maintaining prolonged behavior changes are often difficult tasks. Mental health is one of the largest health concerns globally, including for college students.

**Objective:**

Our aim was to conduct an exploratory feasibility study of local community-based interventions by developing Quokka, a web platform promoting well-being activity on university campuses. We evaluated the intervention’s potential for promotion of local, social, and unfamiliar activities pertaining to healthy habits.

**Methods:**

To evaluate this framework’s potential for increased participation in healthy habits, we conducted a 6-to-8-week feasibility study via a “challenge” across 4 university campuses with a total of 277 participants. We chose a different well-being theme each week, and we conducted weekly surveys to (1) gauge factors that motivated users to complete or not complete the weekly challenge, (2) identify participation trends, and (3) evaluate the feasibility of the intervention to promote local, social, and novel well-being activities. We tested the hypotheses that Quokka participants would self-report participation in more local activities than remote activities for all challenges (Hypothesis H1), more social activities than individual activities (Hypothesis H2), and new rather than familiar activities (Hypothesis H3).

**Results:**

After Bonferroni correction using a Clopper-Pearson binomial proportion confidence interval for one test, we found that there was a strong preference for local activities for all challenge themes. Similarly, users significantly preferred group activities over individual activities (P<.001 for most challenge themes). For most challenge themes, there were not enough data to significantly distinguish a preference toward familiar or new activities (P<.001 for a subset of challenge themes in some schools).

**Conclusions:**

We find that local community-based well-being interventions such as Quokka can facilitate positive behaviors. We discuss these findings and their implications for the research and design of location-based digital communities for well-being promotion.

## Introduction

It is not an exaggeration to say that mental health is one of the most significant issues of our time, and wellness has never been more topical than it is today. Mental health conditions account for one-third of adult health conditions, and suicide is the leading cause of death among people 15-29 years of age [1]. With the continuing shortage of mental health professionals [2-7], it is becoming increasingly clear that the current model of treating mental illness does not sufficiently address the scale and severity of the mental health crisis [1]. It is worth considering how we can augment the traditional medical model of treating mental health conditions with solutions that integrate preventive methods and exploit the ubiquity of technology in positive ways.

Current digital solutions to address behavioral and mental health concerns are often initiated by the individual, such as meditation apps [8,9], fitness apps [10,11], apps providing therapies for developmental delays [12-15], and wearable therapeutics [16-24]). Digital interventions initiated by the individual often require at-home use. When digital solutions are not involved, treatments for behavioral and mental health require repeated in-person visits with a health professional. Although these approaches are helpful, they either do not address the individual’s true needs or are inaccessible to the broader population due to cost and time constraints [25].

Although individuals know that physical activity, nutrition, and sleep, for example, are fundamental components of a healthy lifestyle, this knowledge does not necessarily mean that healthy habits are easy to maintain. Behavior change, particularly as it pertains to health, requires understanding of *why* a change must be made and *how* to actually make the change [26]. Education, knowledge, and awareness are only a few necessary components to encourage behavior change. It is essential to understand how to design and implement behavioral programs and interventions that go beyond these factors to empower individuals to adopt and maintain healthier lifestyles.

Theories of behavior change, termed behavior change theories (BCTs), suggest that intervention effectiveness may be increased through the incorporation of social and cultural factors that also influence behavior [27-29]. These theories targeting lifestyle focus on learning and decision-making to drive action and reflection; however, understanding other factors, such as individual beliefs, motivations, and the environment, are important for continued maintenance of health as well [30,31]. Examples of BCTs that examine these additional factors as applied to health outcomes include the health belief model (ie, behavior change is posited on barriers, benefits, self-efficacy, and threat) and the theory of planned behavior (ie, actions are driven by behavioral intent, subjective norms, and perceived behavioral control) [31-33]. Several of these theories have been studied in the university setting, which is especially pertinent given the Quokka setting. Quokka builds upon prior works by incorporating social, cultural, and local environmental elements into its framework and examining the effects of these community factors on individual action and reflection. Furthermore, Quokka uses several digital intervention techniques (including option-based, attribute-based, and goal-based techniques) that build upon these theories to drive further habit formation and maintenance [30].

There is a strong, well-researched connection among social influence, social media, and health and wellness [34,35]. Health habits are influenced by peers and within social networks. College student health and wellness occupies a particularly interesting and pressing niche, partly due to the prevalence of mental illness in the college population [36-38] and partly due to the unique confluence of communities, resources, and types of development represented during this formational stage of life [39]. College students are uniquely bound to their local and social communities, and their health is largely influenced by both these pivotal factors during their time as students and by the experience of caring for their health independently (eg, determining their own course of physical activity and diet without the aid of others). Committing to change habits as part of a group, such as one’s college peers, can increase the odds of success due to the communal experience and accountability that comes with social pressures. Because of the consistent and ubiquitous prevalence of mental health and other wellness issues across school campuses [40,41], we were interested in exploring the potential of social technologies for behavior change specifically within networks of college students.

Social technologies, both existing platforms and domain-specific technologies, have been used to advance behavior change related to health. A Social-Local-Mobile (So-Lo-Mo) app has been developed to help addicts quit smoking [42]. Twitter has been considered and studied as a platform to disseminate public health information and has successfully changed the attitudes of tweet recipients [43], which is the first step toward successful behavior change. Social influence has been documented to drive engagement in web-based health applications [44]. For example, community programs hosted on digital platforms have facilitated behavior change to increase walking [45] and self-manage diabetes [46].

The idea of digital interventions that feature an online community to aid behavioral change outcomes is not novel. Examples include the AFFIRM Online program [47], Facebook groups for connecting populations [48], and targeted messaging on social media platforms [49]. There are also existing digital interventions that use a local community and local resources to facilitate behavior change. Examples include the Atmiyata intervention approach [50], SocialNet [51], and the +Connect intervention [52]. These prior social technologies do not incorporate a local community aspect into the online social community.

In contrast to these prior works, we test the feasibility of a mental health digital intervention that leverages both local health opportunities and community-based programming to drive behavior change in a single social network. Toward this end, we developed a web platform, Quokka, that promotes an interventional program, the Quokka Challenge. The challenge capitalizes on the established success of community-based social programs for behavior change via a digital intervention. We expand upon the successes of prior literature by exploring the incorporation of the physically local community into a social digital intervention. We note that the primary goal of this study is not to provide a controlled trial or to claim that Quokka has been fully evaluated as an intervention. Instead, our goal is to test the feasibility of such a system by verifying that study participants engage in the behaviors suggested by Quokka for the duration of the program.

The Quokka system was used during the Quokka Challenge, which took place during the fall academic quarter/semester of 2017 and served as a feasibility study for digital well-being programs focused on local, social, and novel experiences. We provide a description of the challenge themes provided each week and describe a longitudinal 6-to-8-week remote pilot feasibility study we ran on 4 independent college campuses. We analyze user retention and participation and code responses to free-form surveys administered to participants at the end of each challenge week. We end with a discussion of the effects of social connectivity, importance of community, limitations of the study, and future directions.

### The Quokka Platform and Challenge

#### Overview

We designed the Quokka Challenge, hosted on the Quokka web platform that we designed and developed, as a new program in the fall academic quarter/semester of 2017 to promote healthier habits in the university setting. The design and implementation of the program were influenced by prior research in the field, although it was uniquely created for the university setting. This manuscript highlights Quokka’s first pilot programs, evaluating its framework’s potential for increasing participation in healthy habits.

Three social elements are fundamental to Quokka’s program: culture, competition, and community. The goal of the program is to change health behaviors by making habit-building easier, more fun, and more social. To do this, the program uses (1) network tools (eg, existing cultures, clubs/social groups) to leverage social influence, (2) gamification (eg, competition, trophies) for intrinsic motivation, and (3) events and external rewards (from local businesses, resources, and student clubs) to further motivate participation and create a sense of confluence for users within communities that are practicing the same habits at once.

The Quokka Challenge follows the same series of user flows and prompts, regardless of where the program is run. Each week, users receive a “Challenge of the Week” email in their inbox. Included is the challenge itself, scientific research supporting the habit, and a list of suggested resources, both remote and local.

#### Quokka System

**Figure 1:**
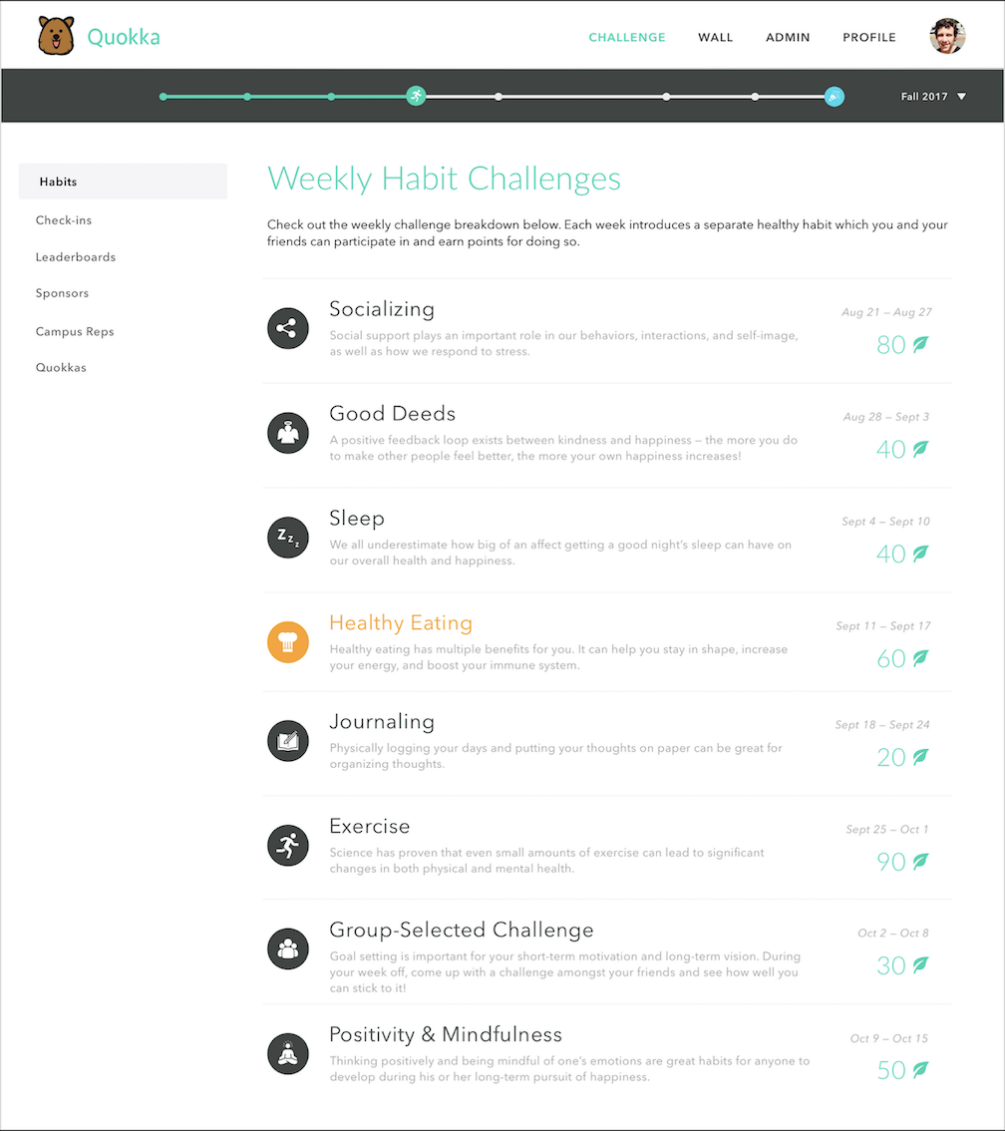
Example user view of weekly challenges. Users see a progress bar at the top of the page visualizing challenge progress. Weekly challenge habits for the entire challenge are shown to users, along with the dates of the challenge, allowing users to plan ahead.

**Figure 2:**
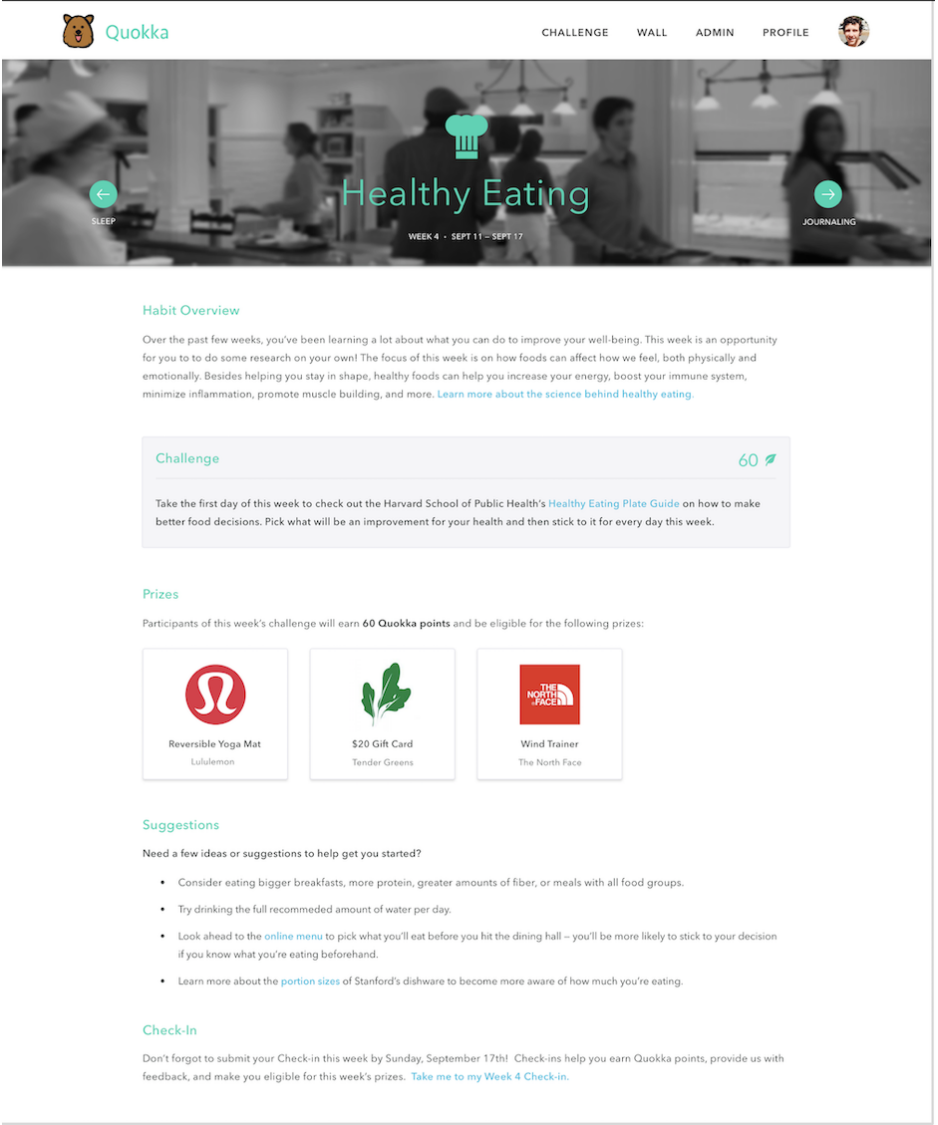
Example user view of a particular challenge, in this case "Healthy Eating." An overview of the challenge (including further research details), the exact challenge, the prizes awarded for completing the challenge, suggestions for particular activities to complete the challenge, and instructions for providing study feedback are displayed.

The Quokka system consists of a website that provides information about challenge themes through a new community-based social network. On the Quokka website, users see a progress bar at the top of the page visualizing their challenge progress (Figure 1). Weekly challenge habits for the entire challenge are shown to users along with the dates of the challenge, allowing users to plan ahead. Users can read an overview of the challenge, including further research details, the exact challenge, the prizes awarded for completing the challenge, suggestions for particular activities to complete the challenge, and instructions for providing feedback (Figure 2).

**Figure 3:**
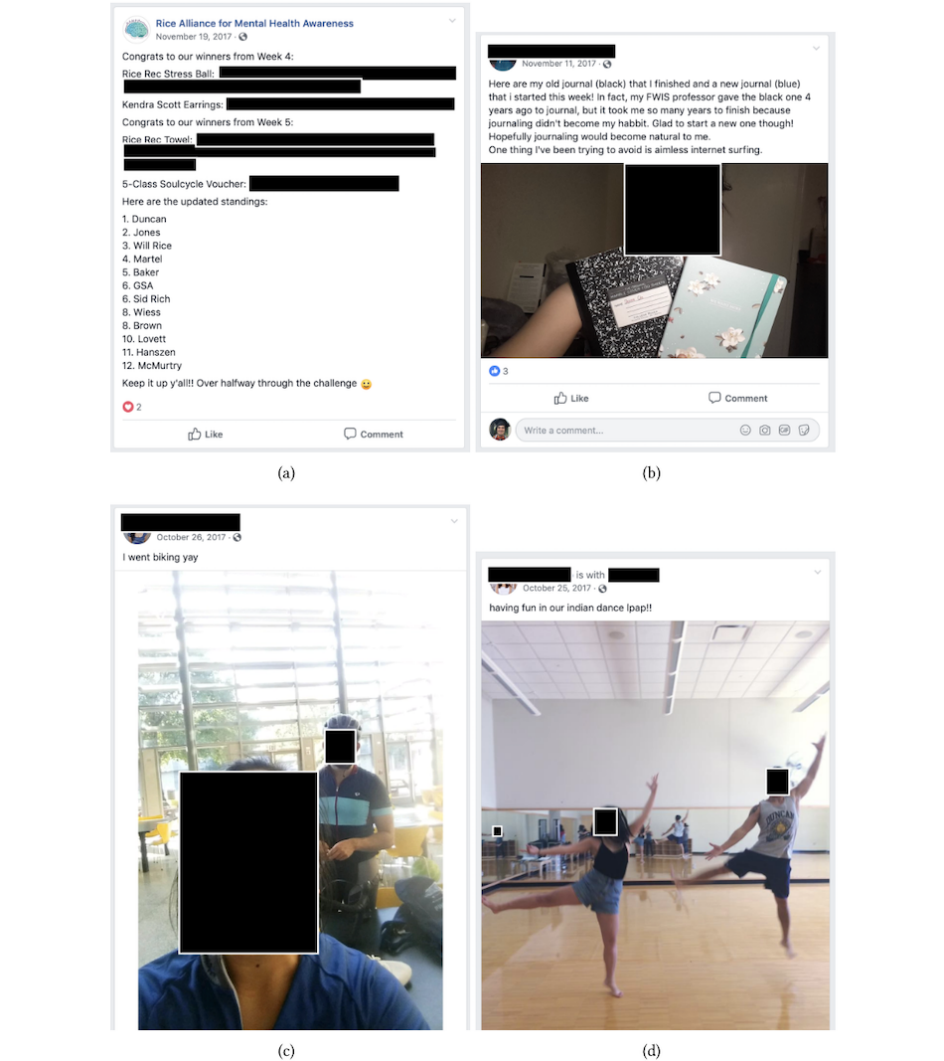
Each university program created Facebook event pages for participants to share their experiences with peers. (a) The coordinator from the university posts regular updates to the Facebook page, including university-specific prizes and rankings. (b) Individual users share updates on the Facebook event page, often garnering reactions from other challenge participants. (c) and (d) Friends can participate in activities together and share on the event page.

An additional aspect to the Quokka system is the option for participation by sharing photos on a community Facebook page hosted by a club at their university (Figure 3). This encourages social participation and is relevant as another program component facilitating the local and social drivers of the program.

Toward the end of the week, users are prompted, via an email notification, to submit a check-in by answering a set of provided questions. Users who answer the set of check-in questions are then entered into a pool of participants eligible to win a prize. Prize winners each week are randomly selected from this pool. Optionally, users can attend (in-person) events that are related to the week’s theme. These events are often hosted in partnership with university health resources, student clubs, and local sponsors.

### Challenge Themes

It is critical to provide health-related educational information to motivate behavior change. We hypothesized that the more that people understand the reasons why a behavior is generally considered healthy, the more motivated they will be to engage in that healthy behavior. Each recommendation made to participants from the challenges was supported by documented research and resources to connect students to more information about health that provides knowledge and motivation regarding the challenge and overall wellness. This scientific research (the “why”) supplemented the challenges (the “what”) as well as the set of resources and tools (the “how”) that could be used to help users successfully complete each challenge.

Each local program selected their weekly “challenge themes” from a set of eight options, with different language provided to users for each theme (Table 1).

**Table 1:** Weekly challenge themes for the eight challenges of the Quokka program and accompanying messages.

Theme	Message
Socialize	“Take 3 purposeful study breaks this week to talk to friends. Schedule a call with someone you haven’t talked to in a while, put away your phone during meals with others, or invite friends to join the Challenge and keep you on track.”
Exercise	“1. Try 3 different types of exercise this week. 2. Move More! Sit less, walk or bike instead of driving, and take stairs over elevators.”
Good Deeds	“Pick a good deed to do or help a person every day this week. Doing something nice, like buying someone coffee or bringing soup to a sick friend, might encourage them to spread the cheer, too!”
Healthy Eating	“1. Add More (of the ‘good’ things) – more water, more fruits and vegetables, and more whole grains to your meals. 2. Be more mindful – of how much you’re eating and when you’re full, of when you’re mindlessly snacking, or of how you feel after you eat different foods.”
Journaling	“Pick a time to journal every day this week – right when you wake up, as an afternoon study break, or before you head to bed. Be consistent, even if it’s just for a few minutes every day.”
Sleep	“Get a full night’s sleep every day this week. Try to keep a consistent schedule and log how many hours you actually sleep and how you felt as a result.”
Positivity/Mindfulness	“1. Practice positive thinking and be kind to yourself and to your peers. To wrap up this week, make a purposeful effort to give out positive comments and compliments to your friends-verbally or written. 2. Acknowledge negative thoughts, but don’t dwell. See how you can reframe your perspective whenever you experience a negative thought this week.”
Campus-Selected Theme	Each campus had the option to select one of the following messages: Giving Thanks: “1. Write thank-you notes. Think of people around you who you don’t stop to thank enough. Perhaps it’s close friends who look out for you/have your back, or perhaps it’s people who play a smaller role in your life – dining hall staff, administrators, people who open doors for you... 2. Keep a gratitude journal. Write down a few things you’re thankful for every day. If it’s a loved one, choose to share and let them know.” Self-Care: “1. Every day, take at least 30 minutes to do an activity that you love! Pick something that makes you feel good, and that you don’t usually make time for! It can be watching an episode of your favorite TV show, reading a book while drinking tea, drawing, or painting a picture. 2. Work on an important relationship at least once this week. Let someone in your life know what you need from/in your relationship with them. Let someone know why you are thankful for them. 3. At least once this week, talk to a friend about how you are coping with work and life demands.”

## Methods

### Recruitment

We reached out to over 15 US colleges and universities and met with several administrative health services and student health club staff members to discuss the possibility of running a program on their campuses. Because this was an early pilot, we chose a small subset of schools (Duke University, University of North Carolina at Chapel Hill [UNC], Rice University, and Tufts University) to coordinate programs based on their overall interest and availability to dedicate time and effort toward participating. Typically, one student health club or group per campus would become the designated “host” while working with other school resources and groups to customize their programs.

Coordinators at each university were responsible for the enrollment of participants in the 2-week period leading up to the Quokka Challenge start date. Coordinators used school email listservs, Facebook event pages, and on-campus recruiting efforts to garner interest.

### Study Design

Of the eight potential challenge themes, university coordinators chose the subject and order of each week for their respective challenges. While the Duke University and UNC coordinators organized 6-week Quokka Challenge programs, the Rice University and Tufts University coordinators opted to include 2 additional weeks, for a total of 8 weeks. The challenge theme order for each university was as follows:

- Duke University (6 weeks): Socializing, Healthy Eating, Exercise, Good Deeds, Sleep, Self-Care
- UNC (6 weeks): Healthy Eating, Exercise, Socializing, Self-Care, Sleep, Positivity/Mindfulness
- Rice University (8 weeks): Healthy Eating, Exercise, Good Deeds, Journaling, Sleep, Give Thanks, Socializing, Positivity/Mindfulness
- Tufts University (8 weeks): Socializing, Exercise, Good Deeds, Healthy Eating, Journaling, Self-Care, Sleep, Positivity/Mindfulness

The program focused on one habit per week, although participants were encouraged to adhere to whichever habits they found to be the most effective throughout the duration of the program. A final survey was sent to participants at the end of each program to collect input and feedback from them, and respondents were asked to cite which habits they had continued and were planning to continue from then on, although this was not further assessed after the program completion.

Every week, we sent a check-in email to all study participants toward the end of the weekly challenge. These check-ins consistently asked the same set of questions: (1) “What’d you do to complete this week’s challenge?” (2) “Tell us about your experience. Did you enjoy it or notice anything different about yourself?” (3) “Any additional comments (about the week or the overall challenge)?”

For this study, we tested the following hypotheses:

- H1: Quokka participants will self-report participation in more local activities than remote activities for all challenges.
- H2: Quokka participants will prefer social activities over individual activities for all challenges.
- H3: Quokka participants will prefer new activities over familiar activities.

We note that we present a feasibility study of social community–based wellness interventions, and the above hypotheses are therefore exploratory in nature.

### Facebook Event Page

Each university program had its own Facebook event page where participants could post shared experiences with others. We used Facebook in lieu of reimplementing a social network within the Quokka interface to maximize the reach of coordinator messages. Figure 3 shows example posts from one of the Facebook event pages.

### Role of Coordinators

Volunteer coordinators at each campus had responsibilities that included (1) enrolling participants in the program, (2) meeting with their self-organized committees regularly, (3) customizing and sending emails through our website, (4) organizing and hosting related challenge events, (5) establishing relationships with local businesses and resources, and (6) securing and distributing prizes to weekly challenge winners. Although the same program content was shared with all the coordinators, they were responsible for the tailoring of their own prizes, events, and outreach.

### Question Coding

To understand the impact of the challenge theme on behavior and to tease apart differences in effects between schools, we qualitatively coded all weekly check-in responses from all study participants into the following categories: (1) whether the challenge for that week resulted in activities that were local or remote, (2) whether the challenge resulted in group or individual activities, (3) whether the challenge created a familiar or new habit, and (4) whether the challenge resulted in a positive, negative, or neutral experience. Coding of questions was performed by 3 independent raters recruited on Upwork, a popular web-based freelancing platform that connects workers to job providers. To reach the final category, a majority-rules consensus was taken for the categorical labels provided by raters. In cases where all 3 raters disagreed, the authors provided the final rating. Protected user data were anonymized when provided to Upwork workers.

### Statistical Tests

To perform statistical testing for H1-H3, we conducted a binomial proportion test in which we used the proportion of local (H1), social (H2), and new (H3) self-reported activities per week. The null hypothesis was that the proportion would be 0.5 (equal numbers of local and remote, social and individual, as well as new and familiar activities). The goal was to determine if the increased rates of local, social, and new self-reported activities were statistically significant. We calculated a Clopper-Pearson binomial proportion confidence interval for one test; this method leverages the cumulative probabilities of the binomial distribution.

## Results

### User Statistics

Across the 4 schools, a total of 1038 people signed up during the 2-week enrollment period leading up to the challenge. Of that total, 277 users completed a week 1 check-in survey; this constitutes the total number of participants who were evaluated during this study. While the Duke University and UNC coordinators organized 6-week Quokka Challenge programs, Rice University and Tufts University opted to include 2 additional weeks for a total of 8 weeks. Due to the differences in duration, we have listed the total number of users who submitted a check-in during their respective “final” weeks, which occurred at either the 6- or 8-week mark (Table 2).

According to the US Census [53], typical demographics of college students in the United States follow an approximate equal split of women and men (with women holding a slight majority). The vast majority of these students are between 18 and 24 years of age (87.5% in 2017, when this study took place). By ethnicity, the US undergraduate college student population in 2017 was approximately 53% non-Hispanic White, 21% Hispanic, 15% Black, 8% Asian, and 3% non-Hispanic “other.”

For the 4 universities included in this study, the typical demographics of their undergraduate college students followed a similar pattern to the national statistics: approximately equal split between women and men, age primarily between 18 and 24 years, and predominantly identifying as non-Hispanic White, with varying distributions of students identifying as Hispanic, Black, Asian, or “other.”

**Table 2:** User retention across the 4 universities participating in the study, according to the respective 6- or 8-week durations of the program.

School	Program duration (weeks)	Users who initially signed up, n	Users who completed week 1 check-in, n	Users who completed final check-in, n	Retention (%)
Duke University	6	144	36	17	47.2
Rice University	8	491	125	28	22.4
Tufts University	8	153	61	13	21.3
University of North Carolina at Chapel Hill	6	250	55	11	20

### Evaluation Outcomes

All 3 hypotheses were confirmed: significantly more local than remote (H1, Table 3), group than individual (H2, Table 4), and familiar than new (H3, Table 5) activities were reported by participants across all challenges. P values from a Clopper-Pearson binomial proportion confidence interval for one test are included in the tables. After Bonferroni correction, we rejected the null hypothesis that similar proportions of users would participate in local and remote activities during the challenges (Table 3). Instead, there was a strong preference for local activities for all challenge themes. Similarly, users significantly preferred group activities over individual activities (Table 4). For most challenge themes, there were not enough data to significantly distinguish preferences toward familiar or new activities (Table 5).

The challenge theme had a noticeable effect on the count of users who reported whether the week’s challenge included local versus remote activities (Table 3), individual versus group activities (Table 4), and familiar versus new activities (Table 5). This indicates that the challenge theme had a strong effect on the engagement of the participants. Interestingly, the participants’ school did not have a noticeable effect, validating the influence of the challenge theme on the type of behavior regardless of the environment of the participant.

According to the qualitative analysis performed on each school’s complete set of responses, 95.1% of survey responses from Rice University over the course of the challenge exhibited positive sentiment, 92.2% of Duke University survey responses exhibited a positive sentiment, 96.4% of Tufts University survey responses exhibited a positive sentiment, and 92.1% of UNC survey responses exhibited a positive sentiment. Of all 6 possible pairs of schools, a paired Welch *t* test between every survey response between all participants in both schools in the pair showed that there was no statistically significant difference between any pair of schools in the sentiment of participating students (all P>.04) after accounting for multiple hypothesis testing using Bonferroni correction.

**Table 3:** Counts of users who reported local and remote activities across the challenges for all schools. All P values are significant after Bonferroni correction.

School and values		Challenges								
		Socialize	Exercise	Good Deeds	Healthy Eating	Journaling	Give Thanks	Sleep	Positivity and Mindfulness	Self-Care
**Duke University**										
	Local, n	81	36	23	66	N/A^a^	N/A	38	N/A	41
	Remote, n	16	1	1	0	N/A	N/A	0	N/A	1
	P value	<.001	<.001	<.001	<.001	N/A	N/A	<.001	N/A	<.001
**Rice University**										
	Local, n	72	158	88	269	94	71	86	58	N/A
	Remote, n	12	0	0	5	0	1	1	0	N/A
	P value	<.001	<.001	<.001	<.001	<.001	<.001	<.001	<.001	N/A
**Tufts University**										
	Local, n	143	91	48	62	36	N/A	30	28	27
	Remote, n	21	0	1	0	8	N/A	1	0	2
	P value	<.001	<.001	<.001	<.001	<.001	N/A	<.001	<.001	<.001
**University of North Carolina at Chapel Hill**										
	Local, n	40	46	N/A	122	N/A	N/A	26	21	31
	Remote, n	7	2	N/A	0	N/A	N/A	1	0	1
	P value	<.001	<.001	N/A	<.001	N/A	N/A	<.001	<.001	<.001

^a^N/A: not applicable.

**Table 4:** Counts of users who reported individual and group activities across the challenges for all schools.

School and values		Challenges								
		Socialize	Exercise	Good Deeds	Healthy Eating	Journaling	Give Thanks	Sleep	Positivity and Mindfulness	Self-Care
**Duke University**										
	Individual, n	29	30	18	65	N/A^a^	N/A	41	N/A	40
	Group, n	71	5	7	2	N/A	N/A	1	N/A	8
	P value	<.001^b^	<.001^b^	.03	<.001^b^	N/A	N/A	<.001^b^	N/A	<.001^b^
**Rice University**										
	Individual, n	105	163	86	302	99	76	108	68	N/A
	Group, n	43	9	25	0	0	13	0	8	N/A
	P value	<.001^b^	<.001^b^	<.001^b^	<.001^b^	<.001^b^	<.001^b^	<.001^b^	<.001^b^	N/A
**Tufts University**										
	Individual, n	85	98	22	65	48	N/A	34	30	29
	Group, n	98	10	41	3	3	N/A	0	1	11
	P value	.34	<.001^b^	.02	<.001^b^	<.001^b^	N/A	<.001^b^	<.001^b^	.004
**University of North Carolina at Chapel Hill**										
	Individual, n	28	50	N/A	136	N/A	N/A	31	21	34
	Group, n	20	1	N/A	0	N/A	N/A	0	3	3
	P value	.25	<.001^b^	N/A	<.001^b^	N/A	N/A	<.001^b^	<.001^b^	<.001^b^

^a^N/A: not applicable.

^b^Significant after Bonferroni correction.

**Table 5:** Counts of users who reported familiar and new activities across the challenges for all schools.

School and values		Challenges								
		Socialize	Exercise	Good Deeds	Healthy Eating	Journaling	Give Thanks	Sleep	Positivity and Mindfulness	Self-Care
**Duke University**										
	Familiar, n	91	30	17	48	N/A^a^	N/A	39	N/A	40
	New, n	3	6	6	24	N/A	N/A	2	N/A	2
	P value	<.001^b^	<.001^b^	.02	.005	N/A	N/A	<.001^b^	N/A	<.001^b^
**Rice University**										
	Familiar, n	43	34	27	4	3	32	2	18	N/A
	New, n	13	31	13	3	4	2	0	2	N/A
	P value	<.001^b^	.71	.03	.71	.71	<.001^b^	.16	<.001^b^	N/A
**Tufts University**										
	Familiar, n	68	17	19	3	14	N/A	1	0	10
	New, n	6	12	1	0	0	N/A	0	0	1
	P value	<.001^b^	.35	<.001^b^	.08	<.001^b^	N/A	N/A	N/A	.007
**University of North Carolina at Chapel Hill**										
	Familiar, n	29	20	N/A	68	N/A	N/A	15	12	17
	New, n	13	17	N/A	39	N/A	N/A	8	6	10
	P value	.01	.62	N/A	.005	N/A	N/A	.14	.16	.18

^a^N/A: not applicable.

^b^Significant after Bonferroni correction.

### Participation Due to Localized Social Influence

Although many of the challenges were focused on individual execution and adherence (ie, did not require interactions with others in order to “complete” the challenge), many users cited the importance of the simultaneous participation of the broader community. Acknowledging that others at the university were fulfilling the same challenges at the same time, users noted that this phenomenon created “positive peer pressure,” encouraging them to complete each week. Additionally, some users noted that the opportunity to participate with others meant they could also share their own lessons more broadly. For example, a user from Rice during the Healthy Eating week stated, “I take a nutrition/health course that teaches me a lot about physical exercise and the importance of dieting so it was great to be able to find other people doing the Quokka Challenge and help them make better food choices.”

### Shared Experiences

The communal experience among users within the same community offered reminders and nudges, encouraging continued participation. Examples of contributions to these shared experiences were unified event offerings, photo sharing on the Facebook pages, and built-in university and friend networks. For example, one user from Rice University during the Healthy Eating week commented:

Seeing the Color My Plate [activity on Facebook] inspired me to eat healthier because other people were doing it! I am better at sticking to good habits if I know people around me care as well.

The shared experience of the Quokka Challenge also prompted users to cite feeling more connected as a result, with one user from Tufts University during the Socializing week stating:

I had a wonderful experience completing this week’s challenge! I have felt more positive and connected as a result of prioritizing time for relationships.

### Local Community-Supported Resources

Every challenge included a set of community-supported resources for participants to learn more about. These recommendations were tailored for the local community and featured information and tips on using resources such as university programs, student clubs, and nearby businesses. Some programs also included external resources (eg, podcasts, apps) to supplement them. For example, participants could learn more about group fitness classes, fitness assessments, or personal training programs offered at the campus recreation center during the Exercise week. In turn, some users stated plans to continue to use new resources they discovered through the challenge, as exemplified by one user at Rice University who stated, “I discovered more ways to enjoy the good weather and all of the resources offered through the Rec center (spin classes, borrowing equipment, etc.) and know that I’ll try to incorporate them more into my weekly exercise routines!”

### User Reflection

Throughout the challenge, users commented on the effects and impact that participating in the Quokka Challenge had on them. The program encouraged users to practice weekly wellness habits, focusing on simple and manageable ways to engage in behavior change. This helped prepare them for a longer-term commitment to being mindful and extending these behaviors beyond the challenge duration. One Rice user during the Exercise week said:

I was really forced to re-examine my health and exercise routine that I had settled into after 2 months into college. This challenge has inspired me to make small changes to see big results in my health and fitness.

Beyond learning more research and facts about the individual habits highlighted in each week’s challenge, users noted other lessons and outlooks they learned in the process. Users at Rice University, for example, reported that the Quokka Challenge helped them reflect more on the benefits of engaging in well-being–promoting activities. Some of these personal learnings also led to further goal setting and commitments to future self-care, as demonstrated by a Rice user stating:

It made me reflect on my life choices and be more mindful of many of the things I do. It made me change my behavior for the better and be more considerate, both with myself and others.

## Discussion

### Principal Results

Here, we discuss Quokka, a local community-based social network designed to encourage and promote health awareness and behavior change by hosting well-being “challenges” across different college campuses. Participants were encouraged to engage in different behaviors, such as drinking more water, exercising, and journaling, to improve overall wellness. Students were also provided implicit social incentives to participate via the social integration of the challenges, as “challenge coordinators” encouraged participants to engage with one another through email and social media.

By encouraging networks of friends to promote healthy behaviors on campus through Quokka challenges, we aimed to create a positive impact that permeated throughout the entire social network within these communities. We customized the Quokka program to each campus and personalized the program’s components to increase the familiarity, comfort, and connections for participants. We tailored health communications with the goal of helping people feel more motivated by their localized resources and thus more likely to make decisions that will help them achieve their health goals. We further encouraged participation in these resources with incentives, such as prizes and praise. By focusing on short-term, attainable goals (eg, focusing on one healthy habit per week), we compartmentalized healthy habits for busy students to focus their efforts and see results on a small yet consistent scale.

After Bonferroni correction, we rejected the null hypothesis that similar proportions of users would participate in local and remote activities during the challenges (Table 3). Instead, there was a strong preference for local activities for all challenge themes. Similarly, users significantly preferred group activities over individual activities (Table 4). For most challenge themes, there were not enough data to significantly distinguish preference toward familiar or new activities (Table 5).

The retention rate per school ranged from 20% to 47%, with a median retention of 21.5% (Table 2). Two of the schools had a 6-week (42-day) challenge, and two had an 8-week (56-day) challenge. These retention numbers are significantly greater than the average retention rates of 93 well-being apps evaluated in a systematic study, which had a median 15-day retention rate of 3.9% and 30-day retention rate of 3.3% [54]. This suggests that the combination of social community and tailored local experiences created by the Quokka experience created a unique environment that promoted high levels of engagement and retention. A future controlled study is needed to determine whether the tailored local experiences, the social community, or a synergistic combination of these aspects drove the high engagement rates.

### Importance of Community

The community of local businesses can also play a substantial role in contributing to the “health community.” For example, affluent areas with gyms, health food restaurants, yoga studios, etc, are often stereotyped to be “healthier” [55]. Social marketing has been proven to be especially effective in promoting health and igniting healthy behavioral change [56-58], and Quokka capitalizes upon this by partnering with local businesses to offer prizes such as free yoga classes and coupons for healthier food vendors to engage in popular health marketing. This practice also encourages a sense of comfort and familiarity for users in their local environment by connecting them to their health community beyond campus.

While Quokka simultaneously hosts challenges on multiple college campuses, it customizes each program to be catered to each university’s student body and local environment. This is achieved through partnerships with local mental health resources and services available on each particular campus and with local businesses or school-affiliated groups. Research suggests that the environment in which students participate in such challenges can greatly affect their performance and their continuation of these habits beyond the challenges if supported by community health directives and resources [59-62]. A strong community provides an excellent foundation for building health at a macro level [62,63]. The demand for community resources among college students is growing [64-66], and students are sometimes not even aware of these resources, forgoing possible improvements in health and wellness because of a lack of visibility. Quokka highlights these resources for students participating in challenges, which are particular to each campus and local health department per program.

Based on the analysis, across all 4 schools, Quokka’s challenge themes were largely grounded in being local (vs remote), individual (vs in a group), and based on familiar habits (vs new habits). While users were participating in the Quokka Challenge individually, they were immersed in their local environment, used local resources, and were still surrounded by a broader community of fellow challenge participants. They were also primarily building upon fundamental or familiar habits, while some users reported having “new habit” experiences.

### Achieving Balance of Prescriptive and Suggestive Activities

While many health and well-being–related behavior change apps follow a purely *suggestive* model that only recommends potential healthy activities, others follow a fully *prescriptive* model of requiring users to participate in specific activities at specific times. Quokka attempts to leverage the benefits of both paradigms by following a *balanced* model, removing the need for participants to identify their own initial steps to action and enabling them to instead follow a baseline set of activities to participate in the habit and challenge. We note that Quokka is prescriptive with respect to the type of health activity and not the specific activity to follow (eg, a challenge will recommend exercising and request that users attempt to exercise several times within a week, but it will not explicitly state what exercises users should perform or how or when they should exercise). When the barriers to entry for starting activities are lowered or removed (with suggested local activities or resources), participants feel increased motivation.

### Localized Social Challenges Drive Personalization

The community aspect of the Quokka paradigm enables personalization, which, in turn, drives behavior change. Personalization for this program was not based on the individual or on data collected from the individual; rather, personalization was achieved at the local, in-person environment, and community level. This level of personalization enables a higher degree of user privacy, a concern increasingly at the forefront of public attention in technological health care interventions [67].

### Limitations: Quokka System

Despite the many strengths of this study, it has several limitations. The program used “prebuilt communities” by using the existing college culture and environment and focused its participation recruitment on college-aged students, which are factors that could decrease the generalizability of our results. Because the Quokka Challenge was established around the college context, the time of the study was confounded by differing academic calendars (eg, differences in quarter vs semester calendars).

Quokka attempts to use users’ social networks by allowing them to opt into inclusive, school-specific Facebook groups particular to the Quokka Challenge. This method does not directly exploit friend connections already made on the Facebook platform, however, and thus may not necessarily maximize the primary social connections that users have established prior to participating in Quokka Challenges. Another key design obstacle in harnessing social networks for health and wellness purposes is that users often desire different degrees of privacy. This perhaps requires a more meticulous solution in which users are able to be more selective about which people from their overarching social networks are privy to information a user chooses to share about their own goals and progress [68]. Quokka currently allows users to preserve the privacy of their successes and failures. However, this means that Quokka does not yet employ full accountability. Users can curate which successes to share with their network; thus, they can easily hide their failures.

An overwhelming majority of users (>90% for all schools) found the experience to be positive, indicating that the Quokka Challenge is enjoyable for most users. However, we did not gather baseline data on the participants’ sentiments prior to the challenge. Further studies should evaluate the sentiment of participants using a Likert scale in a controlled fashion.

Further research is needed to determine the effect of increased social interactions and accountability on building healthy habits. Potential future enhancements to the program include shifting to a mobile-based application and conducting more holistic, technical evaluations of health as opposed to strictly using self-reported evaluations (eg, using firmware trackers or implementing tracking features within a mobile app). The research could also be extended to evaluate behaviors prior to, during, and after the Quokka Challenge to assess the extent of behavioral change and habit-forming as a result.

### Limitations: Study Design

Our knowledge about Quokka’s ability to increase social, local, or new activities is limited. Because no control condition was provided, we only know that when participating in the Quokka Challenge, users are more likely to engage in social, local, or new activities than individual, remote, or familiar activities, by a large and statistically significant margin.

In addition, our study analysis consisted of a quantitative analysis on coded qualitative data. Although the purpose of this analysis was to fully understand the extent of the participants’ well-being activities without collapsing their responses into static categories, a more ideal data collection pipeline would include a combination of multiple-choice selection options in addition to the free response component.

### Comparison With Prior Work

To identify prior studies discussed here and in the Introduction, we searched for “digital mental health intervention local community,” “digital mental health intervention online community,” “mental health social network,” and “digital mental health intervention local social network” on Google Scholar as well as the *Journal of Medical Internet Research* search page. We selected articles published after January 2015. We found several digital interventions that feature an online community to aid in the behavioral change outcome. Examples include the AFFIRM Online program [47], Facebook groups for connecting populations [48], and targeted messaging on social media platforms [49]. We also found digital interventions that use a local community and local resources to facilitate behavior changes. Examples include the Atmiyata intervention approach [50], SocialNet [51], and the +Connect intervention [52].

In contrast to these prior works, we explored a mental health digital intervention that leverages local health opportunities and community-based programming to drive behavior change. To our knowledge, no digital mental health intervention has incorporated elements of a hyperlocal community and a social network to ground the intervention via recurring and targeted challenges.

### Opportunities for Future Work

Interventions such as Quokka provide a mechanism for eliciting behavior change from distributed participants. To optimize the provided interventions, direct measurement of behavior changes via machine learning [69-76] along with self-reported questionnaires can generate useful multimodal data sets. Feature selection approaches could be applied to such data streams to identify salient behavioral markers [77-81] of mental health, and classifiers for these could be realized via trustworthy and reliable crowdsourced labeling of the incoming data [82-87]. Privacy-preserving methods are crucial for behavioral data collected from interventions such as Quokka that contain easily identifiable protected health information [67]. We envision the presented feasibility study of Quokka as the first step toward a local community-based precision health care approach [88-92] to mental health.

### Conclusions

We present Quokka, a social network that encourages participation in well-being–promoting activities through weekly local, community-based challenges. We hosted organized challenges to the undergraduate population at 4 universities, presenting weekly well-being challenges organized by challenge coordinators. We find that participation in the Quokka Challenge coincides with positive experiences for participants and promotes self-reported well-being activity. The Quokka paradigm presents a promising sociotechnical methodology for motivating communities to collectively practice health and well-being.

## Abbreviations

BCT: behavior change theory
So-Lo-Mo: Social-Local-Mobile
UNC: University of North Carolina at Chapel Hill
